# Short- and long-term follow-up and additional benefits in a sickle cell disease patient experienced severe crizanlizumab infusion-related vaso-occlusive crisis: A case report

**DOI:** 10.3389/fmed.2022.1048571

**Published:** 2022-11-29

**Authors:** Awni Alshurafa, Mohamed A. Yassin

**Affiliations:** Hamad Medical Corporation, Doha, Qatar

**Keywords:** sickle cell disease, crizanlizumab, vaso-occlusive crisis, hemolytic anemia, infusion-related reactions

## Abstract

Sickle cell disease is an autosomal recessive disorder characterized by the presence of sickle hemoglobin that leads to chronic hemolysis and vaso-occlusive crisis. After decades of limited therapy options, crizanlizumab is a humanized monoclonal antibody approved by the Food and Drug Administration (FDA) in 2019 for sickle cell-related pain crises for patients 16 years of age and above. Although rare, infusion-related reactions, including painful crises, occurred in 3% as per the package insert. However, the data on how to deal with such reactions and about further treatment outcomes are limited as most patients stopped crizanlizumab after the reaction. Herein, we report the good outcome of 13 doses of crizanlizumab in a 19-year-old female patient with sickle cell disease on hydroxyurea, despite experiencing a severe infusion-related painful crisis during the second infusion. Additional benefits of crizanlizumab, in this case, were preventing new episodes of acute chest syndrome, quitting chronic narcotics use, and a remarkable improvement in quality of life and overall performance.

## Introduction

Sickle cell disease (SCD) is an autosomal recessive disorder characterized by the presence of sickle hemoglobin, leading to chronic hemolysis, and vaso-occlusive events. Worldwide, an estimated 20–25 million people suffer from homozygous SCD. Acute pain crisis, acute chest syndrome, hepatic sequestration, and splenic sequestration are the most common vaso-occlusive-related events in SCD patients (1–3). Vaso-occlusive complications in SCD are thought to result from microvascular obstruction by increased adhesion of sickle blood cells to the endothelium that may induce vascular changes, including endothelial damage, vasoconstriction, and inflammation that lead to vaso-occlusion (4).

P-Selectin is a cyto-adhesion molecule in resting platelets and endothelial cell granules, and it is expressed on the cell membrane once activated by inflammation and trauma. The upregulation of P-selectin initiates and maintains the binding of sickle red blood cells and leukocytes to the vessel wall, which contributes to the process of vaso-occlusive crises (5, 6).

Crizanlizumab is a humanized monoclonal antibody approved by the Food and Drug Administration (FDA) in 2019 for sickle cell-related pain crises for patients above 16 years of age (7). It binds to P-selectin, which, in turn, will block the interaction between sickle erythrocytes, leukocytes, platelets, and endothelial cells, leading to improved microvascular blood flow (8, 9). However, the package insert notes that 3% of patients who received crizanlizumab had infusion-related reactions but does not provide management guidance. Moreover, most of the reported cases stopped crizanlizumab after the infusion reaction. Herein, we report the short- and long-term outcomes and other benefits in a case of severe infusion-related painful crisis during the second crizanlizumab infusion in a 19-year-old female patient with SCD.

## Case report

A 19-year-old female patient known case of sickle cell disease (hemoglobin SS) with a history of recurrent vaso-occlusive crisis requiring emergency visits and hospital admissions and a history of acute chest syndrome twice in the last 3 years. She was started on hydroxyurea 5 years ago and maintained a dose of 1,500 mg daily. However, it does not reduce the vaso-occlusive crisis severity and frequency and it does not prevent the occurrence of acute chest syndrome despite being a complaint of hydroxyurea.

The patient and family were counseled and agreed to start crizanlizumab in an attempt to reduce pain frequency and severity. She was started on 29 March 2021 with a dose of 5 mg per kg as a loading dose followed by another 5 mg/kg after 2 weeks then monthly 5 mg/kg. The last dose was cycle 13 on 29 May 2022 then stopped as she became pregnant.

The first infusion was uneventful and well tolerated. The patient came to the second loading infusion after 2 weeks in her baseline clinical status. By the end of the 2nd infusion, she experienced body pain mainly in the back and legs, rated as 9 out of 10 in severity. She was hemodynamically stable when checked with serial vital signs monitoring. There was no fever, skin rash, wheezes, or shortness of breath. Laboratory tests showed mild leukocytosis but hemoglobin level and hemolysis markers were in the baseline (Table 1). She was given subcutaneous 5 mg morphine but did not improve. Another 5 mg subcutaneous was added with no benefit. So the patient was admitted for treatment of vaso-occlusive crisis with patient-controlled analgesia (PCA) for 3 days. The patient and family were counseled about the event and they decided to continue crizanlizumab. In the subsequent infusions, she had premedication with acetaminophen and the infusion was extended to 1 h. It was well tolerated without any pain events during or within 24 h of the infusion.

**TABLE 1:** Changes in laboratory results throughout admission.

Laboratory value	Before infusion	After infusion	Day of discharge
WBC (×109/L)	6.1	14.4	5.6
Hemoglobin (g/dl)	11	10.9	10.4
Reticulocytes count (×109/L)	236	249	263
Reticulocytes %	6.4	6.2	6.9
Platelets (×109/L)	217	208	213
Total bilirubin (mg/dl)	17	18	15

During 13 doses of crizanlizumab plus hydroxyurea 1,500 mg daily, she visited the emergency department three times and was hospitalized once for 5 days due to a painful crisis (Table 2). Other additional benefits after starting crizanlizumab were preventing new episodes of acute chest syndrome, quitting narcotics, and improving quality of life and overall performance.

**TABLE 2:** Frequency of emergency department visits (ED), hospitalization, and total days of hospitalization before and during crizanlizumab use.

	Before crizanlizumab		During crizanlizumab
	Dec 2018–Jan 2020 (14 months)	Feb 2020–March 2021 (14 months)	April 2021–May 2022 (14 months)
ED visits	5	6	3
Hospitalization	3	4	1
Total days of hospitalization	21	27	5

## Discussion

Crizanlizumab is a P-selectin inhibitor approved to reduce the frequency of vaso-occlusive pain crises in sickle cell disease patients. It received FDA approval following SUSTAIN study, a multicenter, randomized, double-blind, placebo-controlled, phase 2 trial, which showed that crizanlizumab therapy resulted in a significantly decreased sickle cell-related pain episodes by 45% and increased the median time to the first and second vaso-occlusive pain events. It is administered intravenously over 30 min, once every 2 weeks for 2 doses then every 4 weeks thereafter (10, 11). The most commonly reported side effects are nausea, abdominal pain, arthralgia, headache, back pain, and fever (12).

Post marketing, cases of severe infusion-related reactions, including severe pain events, have been reported, especially during the first and second infusions, some cases even required hospitalizations. The management of infusion-related pain events in the reported case was to hold infusion, and to give paracetamol, non-steroidal anti-inflammatory drugs, opioids, intravenous hydration, and/or oxygen supply (13–15).

The mechanism of crizanlizumab infusion-related pain crisis is not clear yet. Karkoska et al. suggested that this reaction may be a complement activation-related pseudoallergy (CARPA) as a work-up revealed a mild increase in sC5b-9 level in a 17-year-old male who experienced severe pain in his back and lower limbs within 10 min of the infusion, later he developed fever and hospitalized for 7 days to control his pain then patient and the family asked to stop further crizanlizumab doses (15). Li et al. hypothesized that crizanlizumab-associated painful febrile reaction may be IgG-mediated based on two cases that had reactions after a first uneventful infusion without evidence of mast cell activation or increased IgE production (14).

A systematic evaluation of post-marketing reports of pain events during or after crizanlizumab infusion showed that most affected patients (86%) initially developed infusion-related pain events at the first or second infusion, 71% required hospitalization, and the majority recovered within 3 days; however, crizanlizumab was stopped in 82% of patients after pain reaction, so the data about the long-term outcomes are lacking (13). In our case, the patient developed a severe reaction during the second infusion, which required hospitalization for pain management, then further infusions were uneventful and she had a good outcome. Clinicians need to be aware of rare infusion-related reactions to crizanlizumab even after the first uneventful dose. Additional benefits in this case after starting crizanlizumab were no episodes of acute chest syndrome, quitting chronic narcotics use and there was remarkable improvement in quality of life and overall performance, so, this reflects the importance of reporting the real-world data for novel therapeutic agents in SCD (16, 17). To our knowledge, this is the first case to report the long-term outcome of crizanlizumab after a severe infusion-related painful crisis.

In conclusion, considering the limited data, this case report indicates that patients who had crizanlizumab infusion-related pain reactions may benefit from further infusions and have good long-term outcomes; however, further studies are required to confirm this finding.

## Data availability statement

The original contributions presented in this study are included in the article/supplementary material, further inquiries can be directed to the corresponding author.

## Ethics statement

The case was approved by Hamad Medical Corporation Medical Research Center and the patient signed a written informed consent was obtained from the individual for the publication of any potentially identifiable images or data included in this article.

## Author contributions

All authors listed have made a substantial, direct, and intellectual contribution to the work, and approved it for publication.
